# Isoflurane in (Super-) Refractory Status Epilepticus: A Multicenter Evaluation

**DOI:** 10.1007/s12028-021-01250-z

**Published:** 2021-07-20

**Authors:** Henning R. Stetefeld, Alexander Schaal, Franziska Scheibe, Julia Nichtweiß, Felix Lehmann, Marcus Müller, Stefan T. Gerner, Hagen B. Huttner, Sebastian Luger, Hannah Fuhrer, Julian Bösel, Silvia Schönenberger, Konstantinos Dimitriadis, Bernhard Neumann, Kornelius Fuchs, Gereon R. Fink, Michael P. Malter, the IGNITE Study Group, with support from the German Neurocritical Care Society (DGNI)

**Affiliations:** 1 Department of Neurology, Faculty of Medicine, University Hospital Cologne, University of Cologne, Kerpener Straße 62, 50937 Cologne, Germany; 2 Department of Neurology, Universitätsmedizin Berlin Campus Charité Mitte, Berlin, Germany; 3 NeuroCure Clinical Research Center, Universitätsmedizin Berlin Campus Charité Mitte, Berlin, Germany; 4 Department of Anesthesiology and Intensive Care, University Hospital Bonn, Bonn, Germany; 5 Department of Neurology, University Hospital Bonn, Bonn, Germany; 6 Department of Neurology, University Hospital Erlangen, Erlangen, Germany; 7 Center of Neurology and Neurosurgery, Department of Neurology, Goethe-University Hospital Frankfurt, Frankfurt, Germany; 8 Department of Neurology, University of Freiburg, Freiburg, Germany; 9 Department of Neurology, Heidelberg University Hospital, Heidelberg, Germany; 10 Department of Neurology, Klinikum Kassel, Kassel, Germany; 11 Department of Neurology, Ludwig Maximilian University of Munich, Munich, Germany; 12 Department of Neurology, University Medical Center Regensburg, Regensburg, Germany; 13 Cognitive Neuroscience, Institute of Neuroscience and Medicine, Research Center Jülich, Jülich, Germany

**Keywords:** Status epilepticus, Isoflurane, Epilepsy

## Abstract

**Background:**

We aimed to determine the association between seizure termination and side effects of isoflurane for the treatment of refractory status epilepticus (RSE) and super-refractory status epilepticus (SRSE) in neurointensive care units (neuro-ICUs).

**Methods:**

This was a multicenter retrospective study of patients with RSE/SRSE treated with isoflurane for status epilepticus termination admitted to the neuro-ICUs of nine German university centers during 2011–2018.

**Results:**

We identified 45 patients who received isoflurane for the treatment of RSE/SRSE. During isoflurane treatment, electroencephalograms showed no epileptiform discharges in 33 of 41 (80%) patients, and burst suppression pattern was achieved in 29 of 41 patients (71%). RSE/SRSE was finally terminated after treatment with isoflurane in 23 of 45 patients (51%) for the entire group and in 13 of 45 patients (29%) without additional therapy. Lengths of stay in the hospital and in the neuro-ICU were significantly extended in cases of ongoing status epilepticus under isoflurane treatment (*p* = 0.01 for length of stay in the hospital, *p* = 0.049 for length in the neuro-ICU). During isoflurane treatment, side effects were reported in 40 of 45 patients (89%) and mainly included hypotension (*n* = 40, 89%) and/or infection (*n* = 20, 44%). Whether side effects occurred did not affect the outcome at discharge. Of 22 patients with follow-up magnetic resonance imaging, 2 patients (9%) showed progressive magnetic resonance imaging alterations that were considered to be potentially associated with RSE/SRSE itself or with isoflurane therapy.

**Conclusions:**

Isoflurane was associated with a good effect in stopping RSE/SRSE. Nevertheless, establishing remission remained difficult. Side effects were common but without effect on the outcome at discharge.

**Supplementary Information:**

The online version contains supplementary material available at 10.1007/s12028-021-01250-z.

## Introduction

Status epilepticus (SE) is a life-threatening manifestation of seizures. Refractory SE (RSE) is defined as seizure persistance despite treatment with at least two appropriately chosen and adequately dosed antiepileptic drugs (AED). Approximately 31–43% of SE episodes are counted as RSE in retrospective studies [1–3]. Super-refractory SE (SRSE) represents the most severe type of SE, with mortality rates up to 50%, which is nearly twice as high as in RSE [4–6]. The term SRSE is applied in cases of ongoing seizure activity for more than 24 h despite extensive antiseizure treatment including anesthetics. Although epidemiological data for SRSE are lacking, it is not uncommon in centers with expertise in neurointensive care. One can estimate that approximately 8–15% of SE episodes evolve into SRSE [6, 7].

Despite the ongoing need for adequate therapy options in RSE/SRSE, evidence-based pharmacological treatment recommendations from properly sized randomized prospective studies are not available at the moment and are not to be expected in the near future [8]. Volatile anesthetics, such as isoflurane, have emerged as a potential new salvage therapy option in RSE/SRSE [9]. With the advent of the AnaConDa system more than 10 years ago, the long-term application of volatile anesthetics outside of the operating room (OR) became feasible in intensive care units (ICUs) and has been used successfully in many different indications as an alternative to intravenous anesthetics. Nonetheless, sedation with isoflurane outside the OR is still considered off-label use [10]. Although the mechanisms of action of isoflurane are not fully understood, the enhancement of the inhibition of neurotransmitter-controlled ion channels, such as γ-amino butyric acid, glycine, and glutamate in the central nervous system, is discussed as a possible antiseizure effect. Therefore, potential key advantages of isoflurane could be its unique mechanism of action, its lipophilicity with rapid diffusion and distribution in the central nervous system, its short half-life periods (which allows for excellent drug monitoring), and the lack of interactions with other substances. Nevertheless, reports on seizure control and outcome under isoflurane in RSE/SRSE are scarce [9].

Here, we sought to evaluate the association of isoflurane with seizure termination and side effects in treating RSE/SRSE in a multicenter setting of specialized neurointensive care centers.

## Methods

### Patient Cohort

This was a retrospective multicenter study. Participating centers were members of the Initiative for German NeuroIntensive Trial Engagement, a subdivision of the German Neurocritical Care Society, which is a free alliance of German neurologists and neurosurgeons in neurointensive care units (neuro-ICU) who conduct multicenter clinical trials in neurocritical care.

We included all consecutive adult patients from the participating centers with isoflurane treatment for RSE or SRSE from 2011 to 2018. We excluded patients with posthypoxic myoclonic encephalopathy because of the different pathogenic mechanisms involved.

SE was defined according to previous recommendations [11, 12]. The semiology of SE was classified as generalized convulsive SE if bilateral convulsions were observed; all other forms of SE were classified as not generalized convulsive SE. In cases of not generalized convulsive SE with coma ictal, electroencephalogram (EEG) features were required, as proposed earlier [13]. Etiology was classified into acute symptomatic, remote symptomatic, progressive symptomatic, or cryptogenic. If patients had known epilepsy, we grouped them in the absence of an acute provocation as remote symptomatic etiology. The symptom severity of SE was assessed by the established scoring tools of the Status Epilepticus Severity Score [14] and the Epidemiology-based Mortality Score in Status Epilepticus [15].

Isoflurane was applied via an anesthetic conserving device (AnaConDa; Sedana Medical, Danderyd, Sweden), an anesthetic delivery system developed for the administration of volatile anesthetics outside the OR. AnaConDa is a heat and moisture exchanger inserted in the breaking circuit of patients who are mechanically ventilated, connectable to any ICU respirator. The AnaConDa system was used according to the manufacturer’s instructions. To achieve sufficient sedation in general, an end-expiratory gas concentration (Fet) of at least 0.2–0.6 vol% or a minimum alveolar concentration (MAC) of at least 0.25–0.5 of isoflurane is usually required. To maintain deep sedation, and depending on other factors (such as age, comedication, or temperature), higher concentrations may be necessary. Because this is a retrospective study, timing, duration, monitoring, and control of the isoflurane therapy was subject to the clinician’s prescription or local procedural protocols, and hence varied.

SE was assumed to be terminated (responders) if seizure activity stopped and did not reoccur until discharge from the ICU after the termination of isoflurane treatment clinically in alert patients or if the EEG in repetitive or continuous recordings was free of any evidence of ictal activity, according to previously proposed EEG criteria for nonconvulsive SE [13] in intubated patients or those with a persistent altered state of consciousness. An unfavorable outcome at discharge was defined as a Modified Rankin Scale score of 5 or higher.

Side effects of isoflurane were evaluated for the whole study population. Prespecified side effects were defined as isoflurane related if they occurred during therapy or if conditions worsened and made another intervention necessary (e.g., new or increased use of vasopressors during isoflurane therapy due to hypotension).

The study was approved by the Cologne University Ethics Committee (18–029). Because of the retrospective study design, written informed consent was waived.

### Statistics

Statistical analyses were performed using SPSS 23.0 for Windows (IBM Corporation, Armonk, NY). For comparisons of independent categorical data, the *χ*^2^ test or Fisher’s exact test (if less than five items) was performed; for comparisons of independent metrical data, the Mann–Whitney *U*-test was performed. All tests were performed two tailed. *p* values less than 0.05 were deemed significant.

## Results

### Patient Characteristics

We identified 45 patients with RSE/SRSE and isoflurane treatment for SE termination admitted to the neuro-ICUs of nine German university centers from 2011 to 2018. According to the given SE grading, 12 (27%) patients had RSE and 33 (73%) had SRSE. Patient characteristics are summarized in Table 1.

**Table 1:** Patient characteristics

	Age, median (range) (yr)	Female sex, *n*	Existing epilepsy, *n*	Place of residence before, *n*	GCSE, *n*	Etiology of SE, *n*	STESS, median (range)	EMSE, median (range)	LOS, median (range) (d)	Neuro-ICU duration, median (range) (d)	Time in neuro-ICU before starting isoflurane, median (range) (d)	Antiseizure treatments^a^ before isoflurane, median (range)	Use of immune therapies, *n*	Unfavorable outcome at discharge, *n*
All patients (*N* = 45)	61 (18–81)	21	13	43 home, 2 nursing home	20	22 acute,12 remote, 2 progressive, 9 cryptogenic	3 (0–6)	67 (14–138)	30.3 (9.9–251.1)	24.7 (0.4–251.1)	5.0 (0.0–58.0)	5 (2–10)	20	35

An unfavorable outcome was defined as a patient having an mRS score at discharge of 5 or higher

*EMSE* Epidemiology-based Mortality Score in Status Epilepticus, *GCSE* generalized convulsive status epilepticus, *LOS* length of stay, *mRS* Modified Rankin Scale, *neuro-ICU* neuro-intensive care unit, *SE* status epilepticus, *STESS* Status Epilepticus Severity Score

^a^Includes benzodiazepines, antiepileptic drugs, and anesthetics other than isoflurane

Most cases of RSE/SRSE were of acute symptomatic etiology (49%; details for etiologies are given in Supplemental Table 1). Not generalized convulsive semiology was most frequent (56%). The median number of antiseizure treatments, i.e., benzodiazepines, AEDs, and anesthetics, before the administration of isoflurane was 5 (range 2–10).

Benzodiazepines were given in only 16 of 45 patients (38%), mostly lorazepam (*n* = 9), followed by midazolam (*n* = 5), clonazepam (*n* = 2), diazepam (*n* = 1), and oxazepam (*n* = 1). A combination of different benzodiazepines was given in four patients.

Different combinations of AED were administered to all patients. The following AEDs were given in decreasing frequency: levetiracetam (*n* = 42), lacosamide (*n* = 29), valproate (*n* = 25), phenytoin (*n* = 22), clobazam (*n* = 6), phenobarbital (*n* = 6), lamotrigine (*n* = 6), oxcarbazepine (*n* = 2), topiramate (*n* = 2), vigabatrin (*n* = 1), and brivaracetam (*n* = 1).

Anesthetics before isoflurane were given in 33 of 45 patients (73%) in the following decreasing sequence: propofol (*n* = 20), midazolam (*n* = 20), thiopental (*n* = 8), and ketamine + midazolam (*n* = 4). Of 33 patients, 25 (76%) were given more than two anesthetics before the introduction of isoflurane.

Additional therapies were used in 20 patients for seizure remission: ketogenic diet (*n* = 3), magnesium (*n* = 4), and immunotherapies (*n* = 20), such as cortisone (*n* = 17), intravenous immunoglobulins (*n* = 5), and apheresis therapies (*n* = 7).

Isoflurane was started at a median of 5 days (range 0–58 days) after admission to the neuro-ICU.

An unfavorable outcome at discharge was present in 35 of 45 patients (78%), and 15 of 45 patients (33%) died in the hospital. From the surviving 30 patients, 11 were transferred to another hospital and 18 to a rehabilitation clinic. Only one patient was discharged directly to a nursing home after acute in-hospital treatment.

### Seizure Termination After Isoflurane

Across the entire cohort of 45 patients, SE was finally terminated after isoflurane therapy in 23 patients (51%) and in 13 of these same 45 patients (29%) without additional therapy. During isoflurane therapy, the EEG showed no epileptiform discharges in 33 of 41 patients (80%).

A burst suppression pattern (BSP) was achieved in 29 of 41 patients (71%) with EEG during isoflurane treatment. Depending on what information was available, the achieved minimal and maximal Fet ranged from 0.2 to 3 vol% and the achieved minimal and maximal MAC ranged from 0.1 to 3.5 vol%. Whereas Fet and MAC values above 1 vol% were associated with higher rates of BSP in previous studies, we could not find an association between Fet/MAC values and reaching a BSP in our cohort (*p* = 0.7).

Lengths of stay in the hospital and in the neuro-ICU were significantly extended in cases of ongoing SE under isoflurane treatment (*p* = 0.01 for length of stay in the hospital, *p* = 0.049 for length in the neuro-ICU), whereas the duration of isoflurane treatment was shorter in patients with terminated SE (*p* = 0.03). Detailed information on seizure termination associated with isoflurane is provided in Table 2.

**Table 2:** Association of isoflurane with seizure termination

After therapy with isoflurane	Age, median (range) (year)	Female sex, *n*	Existing epilepsy, *n*	GCSE, *n*	Etiology, *n*	STESS, median (range)	EMSE, median (range)	BSP	Duration of isoflurane, median (range) (d)	Cycles of isoflurane, median (range)	Side effects under isoflurane, *n*	LOS, median (range) (d)	Neuro-ICU duration, median (range) (d)	Time in neuro-ICU before starting isoflurane, median (range) (d)	Antiseizure treatments^a^ before isoflurane, median (range)	Use of immune therapies, *n*	Unfavorable outcome at discharge, *n*
SE terminated (*n* = 13)	65 (22–77)	6	6	6	6 acute, 6 remote, 0 progressive, 1 cryptogenic	3 (1–6)	73 (15–138)	8/11	3 (0–29)	1 (1–2)	12	18.9 (10.9–77.1)	18.9 (10.9–77.1)	3.6 (0.0–21.0)	5 (2–9)	4	11
SE persistent (*n* = 32)	58 18–81)	15	7	14	16 acute, 6 remote, 2 progressive, 8 cryptogenic	3 (0–6)	67 (14–105)	21/30	7 (1–104)	1 (1–3)	28	37.4 (9.9–251.1)	31.9 (0.44–251.1)	5.4 (0.0–58.0)	5 (3–10)	16	24
*p* values	0.2	0.1	0.2	0.9	0.3	0.1	0.3	0.8	**0.03**	0.6	1	**0.01**	**0.049**	0.6	0.8	0.2	0.7

An unfavorable outcome was defined as a patient having a mRS score at discharge of 5 or higher

*BSP* burst suppression pattern, *EMSE* Epidemiology-based Mortality Score in Status Epilepticus, *GCSE* generalized convulsive status epilepticus, *LOS* length of stay, *neuro-ICU* neuro-intensive care unit, *mRS* Modified Rankin Scale, *SE* status epilepticus, *STESS* Status Epilepticus Severity Score

^a^Includes benzodiazepines, antiepileptic drugs, and anesthetics other than isoflurane

### Side Effects of Isoflurane

There were no problems reported regarding the technical implementation of isoflurane treatment, and none of the interventions had to be discontinued for safety concerns. The number of total events was high in the whole study group (40 of 45 patients, 89%). In 40 patients, hypotension occurred during isoflurane treatment (89%). Twenty-two of these patients (55%) needed additional vasopressors. Twenty patients (44%) suffered from a new infection (mainly bronchopulmonary and sepsis). There was one case of paralytic ileus. Clinical and paraclinical outcome data did not differ between groups with and without side effects, as depicted in Table 3.

**Table 3:** Side effects associated with isoflurane

Patients (*N* = 45)	Age, median (range) (year)	Female sex, *n*	Existing epilepsy, *n*	GCSE, *n*	Etiology of SE, *n*	STESS, median (range)	EMSE, median (range)	BSP	Duration of isoflurane, median (range) (d)	Cycles of isoflurane, median (range)	SE remission after isoflurane, *n*	LOS, median (range) (d)	Neuro-ICU duration, median (range) (d)	Antiseizure treatments^a^ before isoflurane, median (range)	Use of immune therapies, *n*	Unfavorable outcome at discharge, *n*
With side effects (*n* = 40)	60 (18–81)	20	12	18	19 acute, 11 remote, 2 progressive, 8 cryptogenic	3.0 (0–6)	70 (15–138)	26/37	6.5 (0–104)	1 (1–3)	12	30.2 (9.9–251.1)	24.4 (0.4–251.1)	5 (2–10)	17	32
Without side effects (*n* = 5)	64 (21–77)	1	1	2	3 acute, 1 remote, 0 progressive, 1 cryptogenic	3 (2–4)	59 (14–73)	3/4	2 (1–20)	1 (1–1)	1	33.1 (18.4–67.8)	32.1 (18.4–54.8)	4 (3–6)	3	3
*P* values	0.9	0.4	1	1	1	0.9	0.1	0.6	0.1	0.2	1	0.9	0.6	0.3	0.6	0.3

An unfavorable outcome was defined as a patient having an mRS score at discharge of 5 or higher

*BSP* burst suppression pattern, *EMSE* Epidemiology-based Mortality Score in Status Epilepticus, *GCSE* generalized convulsive status epilepticus, *LOS* length of stay, *mRS* Modified Rankin Scale, *neuro-ICU* neuro-intensive care unit, *SE* status epilepticus, *STESS* Status Epilepticus Severity Score

^a^Includes benzodiazepines, antiepileptic drugs, and anesthetics other than isoflurane

Follow-up magnetic resonance imaging (MRI) was obtained in 22 patients (49%). In ten of them, MRI was unchanged. From the remaining 12 patients, 7 had decreased acute MRI lesions, whereas 5 had progressive lesions. In three of the patients with progressive MRI lesions, these were associated with the underlying disease (*POLG* gene mutation [*n* = 1], intracranial bleeding in herpes simplex encephalitis [*n* = 1], and diffuse atrophy in anti-NMDAR encephalitis [*n* = 1]), whereas in the remaining two patients (9%), MRI progression was discussed as potentially seizure related or treatment related: one patient with known structural epilepsy due to focal cortical dysplasia exhibited hyperintense MRI lesions on T2-weighted fluid-attenuated inversion recovery in the pulvinar thalami, and the other patient with SE of cryptogenic origin developed generalized cerebral atrophy.

## Discussion

This study is the first to evaluate effects on SE termination and side effects of isoflurane in adults with RSE/SRSE in a multicenter retrospective study. We found a high rate of seizure control during isoflurane treatment. Isoflurane was associated with sustained termination of SE in up to 51% of RSE/SRSE episodes, and 29% were terminated without additional therapy. Isoflurane-treatment-related side effects, mainly hypotension and infection, were common (89%) but did not seem to affect clinical outcome at discharge.

The application of isoflurane was feasible in all neuro-ICUs in our study. None of the treatments had to be discontinued because of procedural problems.

Despite the growing use of volatile anesthetics for sedation in ICUs in general, as well as for patients in the neuro-ICU in particular, data about its ability for seizure control in SE are scarce. A thorough literature review identified 13 studies on adults, all of which were monocentric case reports and case series, with a maximum of nine patients included [9]. Seizure activity ceased in approximately 93% of patients in the adult population within minutes of initiation of isoflurane therapy but showed a tendency to relapse after the cessation of treatment. In their review, Ferlisi and Shorvon [16] reported a nearly 100% seizure control during isoflurane treatment, but relapse occurred in 41% of patients on withdrawal, and treatment was discontinued in an additional 7% of patients because of side effects. To the best of our knowledge, our study is the largest cohort on this topic to date in a multicenter design, thereby also reflecting the (real-life) variability of standard care. Overall, we observed comparable seizure control with isoflurane during treatment and an association of isoflurane with sustained SE termination (51%). If other antiseizure treatments remained unchanged or were reduced, we ascribed the termination primarily to isoflurane in 29% of cases. Although a BSP was not achieved in all patients, our data suggest that this did not affect the antiseizure effect of isoflurane. In previous studies, doses of Fet or MAC respectively ranged from 0.5 (initially) to 2 vol% on average (and sometimes a maximum of 5 vol%) to achieve BSP [9, 17]. Doses in our cohort were mainly somewhat in the lower part of this range, which probably explains the lower rate of BSP. The reasons why no higher doses were chosen for a BSP remain speculative because of the retrospective design of the study. For example, different treatment protocols at the centers, the goal of deep sedation with a cessation of the seizure but intentionally without BSP, or side effects, such as massive hypotension and/or a depression of cerebral perfusion pressure, especially in patients with acute cerebrovascular disease (e.g., subarachnoid hemorrhage [18]), may have limited higher doses. Of note, we could not find higher amounts of side effects or outcome differences in those patients with and without BSP, which is in line with prior reports that the achievement of BSP was not correlated with short-term prognosis [3, 19], but BSP has been assigned to affect long-term prognosis unfavorably [19].

Considering the previously reported data, the responder rate of isoflurane (sustainable termination of SE) in our cohort was below that of midazolam (78%), propofol (68%), and thiopental (64%) [16]. Ketamine in combination with other anesthetics has also been used as a salvage therapy in RSE/SRSE, with promising permanent seizure control rates from 57 to 91% [20–22]. However, it must be mentioned that comparing current isoflurane data to the efficacy of other anesthetics is a delicate task given the marked heterogeneity in terms of patients and methods across all studies.

Previous studies have shown that time latency from the onset of SE to treatment initiation is crucial [23, 24]. Unfortunately, we cannot provide exact data on how long RSE/SRSE lasted when the isoflurane treatment was induced because these data were not sufficiently retrievable from the patient files. As an indirect parameter for SE duration, we chose the number of antiseizure drugs that had been used before isoflurane induction and the time from admission to the neuro-ICU until the start of isoflurane; both did not affect treatment outcome.

Surprisingly, the rate of benzodiazepine treatments in the initial stages of SE in our cohort was two times lower than that in an assessment of SE in German-speaking countries that covered roughly the same time span [25], suggesting that SE treatment guidelines were misapplied in the majority of patients. Because the main purpose of our study was to assess the refractory stages of SE, we cannot provide sufficient data to confirm or exclude such a hypothesis. It is important to emphasize that, in most cases, the initial SE treatment takes place outside the hospital. In Germany, the current treatment is administered at the discretion of the responsible emergency physician on site. The data for this study were taken from hospital records, which collected all available information on preclinical and inpatient treatment. However, we cannot rule out the underreporting of preclinical therapies.

It is also noteworthy that a suspected autoimmune etiology of SE occurred in 9 patients, but 17 patients received corticosteroids as an additional therapy. Unfortunately, we could not find detailed information on this subject in the patient files, but we think that the role of additional therapies in SE, such as corticosteroids, would be worth further investigation.

It must be admitted that, despite a considerable SE termination rate, most patients were discharged with an unfavorable Modified Rankin Scale score. One third of all patients died in the hospital regardless of SE termination. However, this may have been related to the patients’ underlying severe brain diseases rather than the SE itself. It is still noteworthy that only one of the surviving patients was transferred to a nursing home, whereas all remaining surviving patients were transferred to a follow-up hospital, mostly for rehabilitation, so one can expect further improvements on long-term follow-up, as shown previously for patients with RSE/SRSE [26].

The most commonly known side effect of isoflurane is hypotension due to vasodilation (presumably peripheral vasodilation in the microcirculation). Accordingly, hypotension under isoflurane was in 89% of cases the most frequent side effect in our cohort, and vasopressors were needed in 55% of cases. However, this problem also occurs similarly with intravenous anesthetics, such as propofol or thiopental. Compared with earlier reports [17], hypotension and infections were less frequent in our cohort. Possibly, not all complications were attributable to the drug, per se, but were alternatively due to prolonged artificial ventilation, prolonged stay in the ICU, duration of RSE/SRSE, or the underlying disease [17].

In summary, we could not find that side effects affected clinical outcome at discharge. However, it should be mentioned that there is a selection bias owing to the retrospective study design: patients with severe hypotension, (risk of) high intracranial pressure, risk of hyperthermia, respiratory failure, or massive bronchial secretion (with less resorption of isoflurane) must be subjected to a special benefit-risk assessment [10, 18, 27].

It has been suggested that isoflurane may cause morphological cerebral MRI changes, especially in the thalamus, hippocampus, and cerebellum, because of proposed disturbances in brain metabolism in a dose-dependent and/or time-dependent manner [28, 29]. On the other hand, similar peri-ictal MRI changes can also be caused by prolonged seizure activity itself [30]. Therefore, we evaluated follow-up MRI in our patients, if available. From 22 patients with follow-up MRI, we found only 2 patients (9%) with progressive MRI alterations that may have been potentially associated with treatment. These two patients received isoflurane for 14 and 61 days. Similar to the studies mentioned above, changes in cerebral MRI due to prolonged SE, due to polypharmacy, and/or in the context of the underlying disease could not be ruled out. Overall, our data suggest a low risk of isoflurane-induced morphological brain damage, as depicted by MRI.

One limitation of this study is its retrospective nature. Another is the relatively small sample size with heterogeneous data, allowing descriptive analysis only. On the other hand, RSE/SRSE are rare conditions, making randomized prospective studies hardly feasible [31]. Our retrospective multicenter study includes the largest and most representative cohort with isoflurane therapy for RSE/SRSE to date. There might very well have been a selection bias toward more severe cases because all participating centers are specialized referral centers for neurointensive care, and thereby results may not be generalizable to all patients with RSE/SRSE. Because the inclusion period ranged over 8 years, there might have been variations in the local management policies. Further, effects of isoflurane on seizure control are difficult to interpret because of the heterogeneity of previous and simultaneous therapies/agents, variable time points of induction, different underlying etiologies of SE, and duration as well as concentration of isoflurane. Isoflurane is most commonly used late as a salvage agent in the treatment of RSE/SRSE, so potentially better effects due to isoflurane induction at an earlier time point seem conceivable, although it is speculative and warrants further investigation. Unfortunately, we were not able to provide long-term outcome data because this was not the primary intention of our study.

## Conclusions

This is the first multicenter evaluation of the association between isoflurane treatment and seizure control, as well as its side effects in adults with RSE/SRSE. The data suggest that isoflurane may be a feasible and effective therapeutic option in terminating SE sustainably and deserves further study. Side effects, i.e., hypotension and infection, were common under isoflurane, but clinical outcome at discharge was not different without those events.

## Supplementary Information

Below is the link to the electronic supplementary material.

Supplementary file1 (DOCX 14 kb)
