# Supplementary material for: Isoflurane in (Super-) Refractory Status Epilepticus: A Multicenter Evaluation
Source: Neurocrit Care. 2021 Jul 20;35(3):631–9. doi: 10.1007/s12028-021-01250-z (PMC8692280; doi:10.1007/s12028-021-01250-z)
Supplement: Supplementary file 1 — Supplementary file1 (DOCX 14 kb) [file 12028_2021_1250_MOESM1_ESM.docx]

**Supplemental Table 1:** Etiologies in RSE/SRSE

| **Symptomatic (N=36)** |
| --- |
| **Acute symptomatic (N=22)**  Acute cerebrovascular (N=4)  Alcohol abuse/drugs (N=3)  Acute head injury (N=2)  Hyponatremia (N=2)  Acute NS-infection (N=2)  Autoimmune (N=9) |
| **Remote symptomatic 12**  POLG-Mutation (N=1)  Chronic cerebrovascular (N=4)  Chronic head injury (N=1)  Chronic CNS infection (N=1)  Known Epilepsy without provocation (N=5) |
| **Progressive symptomatic (N=2)**  CNS tumor (N=1)  Multiple Sclerosis (N=1) |
| **Cryptogenic (N=9)** |
